# Evaluation of the Falls Management Exercise (FaME) programme implemented across three United Kingdom regions: a prospective cohort study

**DOI:** 10.1186/s12889-026-26893-5

**Published:** 2026-03-06

**Authors:** Michael J. Taylor, Denise Kendrick, Carol Coupland, Victoria A. Goodwin, Helen Hawley-Hague, Claire Hulme, Pip Logan, Fay Manning, Tahir Masud, Dawn A. Skelton, Chris Todd, Jodi Ventre, Elizabeth Orton

**Affiliations:** 1 School of Medicine, University of Nottingham, Nottingham, UK; 2 Centre for Academic Primary Care, School of Medicine, University of Nottingham, Nottingham, UK; 3 University of Exeter, Exeter, UK; 4 National Institute for Health and Care Research, Applied Research Collaboration-Greater Manchester, School of Health Sciences, Faculty of Biology, Medicine and Health, The University of Manchester, Manchester, M13 9PL UK; 5 University of Queensland, Brisbane, Australia; 6 Nottingham University Hospitals NHS Trust, Nottingham, UK; 7 Glasgow Caledonian University, Glasgow, UK; 8 Manchester University NHS Foundation Trust, Manchester, M13 9WL UK

**Keywords:** Falls, Exercise, Older adults, Implementation, Evaluation

## Abstract

**Aims:**

The Falls Management Exercise (FaME) programme, delivered over 24 weeks, reduces falls in randomised controlled trials and ‘real world’ delivery, but some services deliver shorter, adapted FaME programmes. We investigated the ‘real-world’ effectiveness of FaME delivered for 12 or 24 weeks across three UK regions.

**Methods:**

*Design*: prospective cohort study.

*Participants*: 1,601 FaME programme attendees (median age 79.5 years), of whom 873 had follow-up data recorded.

*Setting*: 14 provider organisations in Greater Manchester, Devon and the East Midlands.

*Procedure*: Participant data were collected by FaME providers at baseline and up to 24 weeks later.

*Outcomes*: Timed up-and-go (TUG); Short Falls Efficacy Scale-International (Short FES-I); at least one self-reported fall, and number of self-reported falls, in the past 3 months.

*Analysis*: Univariate before-and-after tests; multivariable multilevel logistic, linear, Poisson, negative binomial regression.

**Results:**

At final follow-up (≤24 weeks), compared to baseline, the odds of a self-reported fall within 3 months (Odds ratio 0.16 [95% confidence interval [CI] 0.10 to 0.25], *p* <.001) and TUG time had decreased (difference-between-paired-medians -1.0 (95% CI -1.50 to -1.0), *p* <.001). Short FES-I scores had not significantly changed. Twenty-four- rather than 12-week programmes were associated with faster TUG times (mean difference -3.31 seconds [95% CI -5.97 to -0.65]) and reduced concerns about falling (mean Short FES-I difference -2.00 [95% CI -3.65 to -0.34], *p* = 0.018), but no significant difference in the odds of a self-reported fall or number of falls within 3 months.

**Conclusions:**

FaME programmes of both 12 and 24-weeks were associated with improved functional mobility and reduced falls. Participants who attended 24-, rather than 12-week, programmes had significantly greater improvements in functional mobility and reduced concerns about falling. Due to follow-up data missingness, the study was not adequately powered to detect differences in falls risk or rate between 12 and 24-week programmes.

**Supplementary Information:**

The online version contains supplementary material available at 10.1186/s12889-026-26893-5.

## Introduction

The risk of falling increases with age, and the associated reductions in strength and balance that occur [1]. Falls in older people are associated with high mortality [2] and costs [3] and are common: around 14 per 100 adults in Western Europe aged over 70 years had a fall requiring medical attention in 2017 [2]. The recorded mortality rate from accidental falls has been increasing year-on-year in England since 2001 and was 13.7 per 100,000 persons per year in 2021-3 [4]. The World Falls Guidelines recommend that fall prevention programmes last a minimum of 12 weeks, and state that programmes longer than this are associated with better effects [5].

The Falls Management Exercise (FaME) programme is a 24-week structured multi-component exercise intervention of supervised classes. FaME is delivered by Postural Stability Instructors (PSIs). PSIs are professionals who have received accredited, specialist training (> 200 h self-directed, online and face to face) on the effective delivery of the exercises and behaviour change components of FaME. FaME includes group classes and home-based exercises [6] for people at intermediate and high risk of a fall [5]. The components of FaME aim to enhance strength, balance, endurance, and flexibility, and improve functional floor and gait skills [7]. In the first randomised controlled trial (RCT) of FaME with women with a history of 3 or more falls in the previous year, or at high risk of a future fall, there were significant reductions in falls and injurious falls and improved ability to retain independence at 1 year follow up [7]. A multicentre RCT demonstrated that FaME can reduce falls rates, improve strength and balance and increase physical activity in men and women who were sedentary and at low to intermediate risk of a fall. It showed the programme to be associated with a greater reduction in falls and increase in physical activity than the Otago Exercise Programme or usual care at one-year follow-up [6, 8]. A subsequent observational study evaluated the ‘real world’ implementation of FaME across nine local authorities in the East Midlands region of the United Kingdom (UK) and showed improved functional mobility, confidence and reduced falls [9]. Some UK providers offer shorter 12-week FaME programmes, but their effectiveness compared with 24-week programmes is unknown. The present paper (a component of our FaLls EXercise Implementation [FLEXI] implementation study) investigated the real-world effectiveness of FaME for participants when it was delivered across three UK regions, and the extent to which programme duration (12 or 24 weeks) influenced outcomes.

## Methods

### Design

A prospective cohort study was conducted in people who undertook FaME programmes delivered in Devon, Greater Manchester and the East Midlands between 2022 and 2023. To be included, participants needed to have attended at least one PSI-supervised class, and a provider organisation needed to have collected baseline and outcome data for them.

### Intervention

The FaME structured programme is delivered by PSIs and combines weekly 1-hour PSI supervised classes with home-based exercise [6]. Advantages of FaME compared to alternative programmes include that it works on all components of fitness, not just strength and balance, it considers both static and dynamic balance, reactive stepping and the emphasis on floor work training [10]. The programme is tailored to the individual and is progressive, becoming more challenging over its duration to ensure principles of progressive ‘overload’ are applied for training effects [9, 10].

FaME was delivered via 14 provider organisations in community settings across the three UK regions. These organisations comprised local AgeUK organisations, leisure services, community National Health Service providers and private providers. The delivery of FaME was often adapted locally by the service providers. Adaptations included removal of backward chaining to regain skills of getting down to and up from the floor, floor exercises and Tai Chi, less focus on home exercise component, and the extent of progression of bands (progression of strength) was reduced.

### Data

Trained PSIs at each organisation collected participant information, which included results of assessments they completed with participants, and collated it using a spreadsheet. The outcome variables were: Timed Up and Go (TUG) [11], a measure of functional mobility and risk of falling (participants were observed by the PSIs and timed while they rose from a chair, walked 3 m, turned, walked back, and sat down again); The Short Falls Efficacy Scale-International (Short FES-I) [12] scale (scores can range from 7 [no concerns about falling] to 28 [severe concerns about falling]), as a measure of participants’ level of concerns about falling whilst doing routine activities (this score was analysed as a continuous variable and was also split into a binary measure separating those with scores ≥ 11, indicating those with high concerns about falling, from the remainder [13]); and whether participants reported having fallen at least once within the past three months (based on responses to questions about how many times they had fallen in this period) and number of falls in the past 3 months. These data were collected at baseline (the first PSI supervised class) and at the end of the FaME programme. For all organisations except one, this was at 12- or 24-weeks post-baseline. For one provider organisation, follow-up times varied across the participant group (see FaME programme duration in the Results). Follow-up assessments were conducted in person.

Likelihood of falling can be affected by a number of variables including age [1], gender [2], ethnicity [14] and deprivation [15]. We therefore collected data for these parameters and recorded age in years; ethnicity according to UK 2021 census categories [16]); and deprivation according to Index of Multiple Deprivation (IMD) 2019 [17] quintile for the participant’s home postcode.

### Statistical analysis

Data were described using means and differences between means with standard deviations for parametric continuous variables, medians with interquartile ranges (IQRs) for non-parametric continuous variables and numbers and percentages for binary variables. For univariate before-and-after tests comparing outcomes at final follow-up with baseline values, Wilcoxon Signed Rank tests were used for continuous outcome variables (as these were non-parametric) and McNemar’s χ^2^ tests were used for binary variables.

FaME durations of 12 and 24 weeks were compared due to these being the programme lengths that were most commonly offered by the provider organisations, and differences in outcomes between programmes of these lengths is unknown. To compare different FaME durations (12 vs. 24 weeks), multivariable analyses were conducted using multilevel logistic, linear, Poisson and negative binomial models as appropriate for the outcome variable (programmes that were not 12 or 24 weeks in length were not included in these comparisons). Participant observations were at level 1, provider organisation was at level 2 and region was at level 3. The model included a variable indicating FaME duration (12- or 24-weeks) and all above-listed covariates (age, gender, ethnicity, IMD). Comparisons were made between the durations (12 vs. 24 weeks) for outcome variables recorded at baseline and at final follow-up (baseline values for the relevant outcome were included in the regression models measuring final follow-up outcomes). Ethnicity was included as a non-ordered categorical variable coded as ‘Unknown’ when missing. For all other variables in the regression analyses, people with missing data were excluded from the complete case analysis. We did two regression analyses for number of falls. Our main analysis included the number of falls that participants had experienced in the 3 months prior to final follow-up as the outcome. We also did an additional analysis for which the outcome was falls rate across the study period (up to 24 weeks). This additional analysis was done to enable the first 12 weeks of falls data to be considered for those in the 24-week programmes. As a sensitivity analysis, a multivariable regression was undertaken using equivalent models to those described above, wherein missing age, gender and IMD values were imputed using multiple imputation (MI) by chained equations. For this, 20 datasets were created before the model estimates were pooled. We also conducted an additional sensitivity analysis that did not include ethnicity in the model, due to the prevalence of missing data for this variable. Post-hoc power calculations were performed to investigate the power achieved for non-significant findings. STATA 17 (Stata Statistical Software, Stata Corporation, TX, USA) was used to conduct the analyses. 

### Funding

Funding was received from the National Institute for Health and Care Research Applied Research Collaboration National Priorities Programme (Healthy Ageing, Dementia and Frailty (NIHR201887)). VG is funded by NIHR Applied Research Collaboration South West Peninsula and is an NIHR Senior Investigator; CT is funded by NIHR Applied Research Collaboration -Greater Manchester and is an NIHR Senior Investigator.

### Ethics

Ethical approval was granted by The Health Research Authority and Health and Care Research Wales (22/PR/0634). This study involved the analysis of anonymised data that had been collected routinely by providers (data controllers) and therefore did not require individual consent. These data were shared with the research team for analysis as part of information sharing provider agreements.

## Results

### Demographics

The 14 provider organisations in the three regions collected routine data at baseline from 1,601 people. Table 1 shows demographic information. Median participant age was 79.5 years (IQR 74.5 to 84.5); 61.2% (*n* = 980) were female, 28.7% (*n* = 459) were male (gender data was missing for the remainder). Ethnicity information was supplied for 35.6% (*n* = 586); 556 (94.9%) of these participants were White, 20 (3.4%) were Asian, three (0.5%) were Black; four (0.7%) had mixed ethnicity and three (0.5%) had an ethnicity categorised as ‘Other’. IMD data were reported for 1311 (81.9%). Of these participants, 11.6% lived in the most, and 15.1% lived in the least deprived quintile areas.

**Table 1:** Demographics of study participants

	All participants *n* = 1,601	Those with follow-up data (12 weeks, 24 weeks or an alternative timepoint) *n* = 873	Those without any follow-up data *n* = 728	Those with 12-week follow-up data *n* = 563	Those with 24-week follow-up data *n* = 351	Those with 12-week or 24-week follow-up data and with covariate information required for multivariate analysis inclusion *n* = 443
Median (Interquartile range)						
Age, median (IQR) Missing (*N*, %)	79.5 (74.5 to 84.5) [167, 10.4%]	79.5 (74.5 to 83.5) [74, 8.5%]	79.5 (74.5 to 84.5) [93, 12.8%]	79.5 (73.5 to 84.5) [66, 11.7%]	79.5 (75.5 to 84.5) [58, 16.5%]	79.5 (73.5 to 83.5) [Not applicable (NA)]
*N* (% by group)						
Region:						
Devon	944 (59.0%)	344 (39.4%)	600 (82.4%)	64 (11.4%)	19 (5.4%)	41 (9.3%)
East Midlands	422 (26.4%)	299 (34.2%)	123 (16.9%)	285 (50.6%)	211 (60.1%)	201 (45.4%)
Greater Manchester	235 (14.7%)	230 (26.4%)	5 (0.7%)	214 (38.0%)	121 (34.5%)	201 (45.4%)
Gender:						
Female	980 (61.2%)	561 (64.3%)	419 (57.6%)	362 (64.3%)	215 (61.3%)	317 (71.7%)
Male	459 (28.7%)	247 (28.3%)	212 (29.1%)	138 (24.5%)	92 (26.2%)	126 (28.4%)
Missing	162 (10.1%)	65 (7.5%)	97 (13.3%)	63 (11.2%)	44 (12.5%)	NA
Ethnicity						
Asian	20 (1.2%)	20 (2.3%)	0 (0%)	20 (3.6%)	13 (3.7%)	9 (2.2%)
Black	3 (0.2%)	3 (0.3%)	0 (0%)	3 (0.5%)	0 (0.0%)	3 (0.8%)
Mixed	4 (0.3%)	2 (0.2%)	2 (0.3%)	2 (0.4%)	1 (0.3%)	1 (0.3%)
White	556 (34.7%)	413 (47.3%)	143 (19.6%)	368 (65.4%)	259 (73.8%)	343 (85.5%)
Other ethnicity	3 (0.2%)	1 (0.1%)	2 (0.3%)	1 (0.2%)	1 (0.3%)	1 (0.3%)
Unknown or missing	1,015 (64.4%)	434 (49.7%)	581 (79.8%)	169 (30.0%)	77 (21.9%)	86 (47.1%)
IMD						
1 (most deprived)	186 (11.6%)	106 (12.1%)	80 (11.0%)	75 (13.3%)	25 (7.1%)	76 (17.2%)
2	292 (18.2%)	137 (15.7%)	155 (21.3%)	66 (11.7%)	33 (9.4%)	64 (14.5%)
3	296 (18.5%)	151 (17.3%)	145 19.0%)	77 (13.7%)	46 (13.1%)	82 (18.5%)
4	296 (18.5%)	178 (20.4%)	118 (16.2%)	106 (18.8%)	82 (23.4%)	111 (25.1%)
5 (least deprived)	241 (15.1%)	151 (17.3%)	90 (12.4%)	100 (17.8%)	74 (21.1%)	110 (24.8%)
Missing	290 (18.1%)	150 (17.2%)	140 (19.2%)	139 (24.7%)	91 (25.9%)	NA

### FaME programme duration

Data were collected for 563 (35.1%) participants at 12-week follow-up (this included participants of both 12- and 24-week programmes) and 351 (21.9%) at 24-week follow-up (participants of 24-week programmes only. See Table 1). Data were collected for at least one of these timepoints (i.e., 12 or 24 weeks) for 594 participants.

Of the 14 provider organisations, four only had follow-up information for those who had participated in a 12-week programme (*n* = 98), two only had this for those who participated in a 24-week programme (*n* = 215) and six had this for those who had participated in either a 12- or a 24-week programme (*n* = 281). Two further provider organisations did not have follow-up data at 12- or 24-weeks. Across the provider organisations, there was a total of 243 people (at 10 provider organisations) who participated in a 12-week and 351 (at eight provider organisations) who participated in a 24-week programme and for whom follow-up data were recorded.

One of the 14 provider organisations, which collected follow-up data from 279 participants, collected follow-up information on different dates for different persons rather than at standardised 12- or 24-week follow-up times (the median follow-up time for attendees of this organisation who were reported to have completed their programmes was 14.9 weeks [interquartile range 12.9 to 16.9, inter-decile range 11.3 to 19.1]). Data from these 279 participants were included in univariate tests comparing values at final follow-up with baseline values, but not multivariable analyses as these included a variable denoting whether the programme duration was 12 or 24 weeks. Therefore, a total of 873 (594 plus 279) participants (54.5% of those with data at baseline) had follow-up data reported (see Table 1).

### Univariate analyses

Before-and-after tests indicated that, at the end of a FaME programme, compared to the beginning, a significantly lower proportion of participants (19.3% rather than 47.8% (odds ratio [OR] 0.16, 95% confidence interval [CI] 0.10 to 0.25), see Table 2) reported having fallen at least once in the past 3 months. The before-and-after tests also indicated that participants’ TUG scores at final follow-up were significantly faster than they had been at baseline, with the median of all end-of-programme scores being 8 s (IQR 2–14), compared with 9.5 (IQR 3–17) at baseline. There was no significant change in Short FES-I scores, or the odds of having a Short FES-I score ≥ 11 (greater concerns).

**Table 2:** Descriptive statistics and paired significance tests for timed up and go, Short FES-I and falls in past three months for those who had any final assessment (whether at 12 weeks, 24 weeks or a different time)

Measure	No. of respondents		Median score (Interquartile range), or number (percentage)			Odds ratios (95% confidence intervals) for proportion outcomes; difference between paired medians (rank-based 95% confidence interval) for scale outcomes	*p* values from paired tests comparing baseline and final followup**
			Baseline				
	Baseline	Final follow-up* (proportion of baseline *n*)	All	Those with data at final follow-up	Final follow-up		
Short FES-I ≥ 11	303	142 (46.9%)	119 (39.3%)	82 (57.8%)	72 (50.7%)	0.58 (0.28 to 1.17)	0.14
Short FES-I score	303	142 (46.9%)	12 (9–15)	12 (8–15)	11 (8–14)	-0.5 (-1.0 to 0.0)	0.09
One or more falls in past 3 months	713	389 (54.6%)	362 (50.1%)	186 (47.8%)	75 (19.3%)	0.16 (0.10 to 0.25)	< 0.001
Timed up and go (seconds)	1091	596 (54.6%)	10 (3–16)	9.5 (3–17)	8 (2–14)	-1.0 (-1.50 to -1.0)	< 0.001

*Including only those who also had baseline values reported

**Wilcoxon Signed Rank tests were used for timed up and go; McNemar’s χ^2^ tests were used for the binary Short FES-I and falls outcomes

### Multivariable analyses

Of the 1,601 participants with baseline data, 443 (27.7%) had sufficiently complete information for inclusion in one or more of the complete case multilevel analyses (See Table 1 for characteristics of this population, Table 3 for data completeness information, and Table 4 for analysis findings). For analyses comparing final follow-up measures between 12- and 24-week programme durations, 98 were included in the analysis of Short FES-I scores, 237 in analysis of falls and 277 in analysis of TUG (see Supplementary Table 1). For individuals included in the multilevel analyses, at the end of the programme, those who had attended a 24- rather than 12-week programme had significantly faster TUG (difference between means − 3.31 s [95% CI -5.97 to -0.65], *p* < 0.015), had a lower odds of reporting concerns about falling (odds ratio for Short FES-I score ≥ 11: OR 0.12 [95% CI 0.02 to 0.69], *p* < 0.017), and had a lower Short FES-I score (difference between means − 2.00 (95% CI -3.65 to -0.34), *p* = 0.018). Although there was a reduced odds ratio no significant difference was found between the 12-week and 24-week programmes in the odds of a reported fall in the previous 3 months (OR 0.40 [95% CI 0.12 to 1.26], *p* = 0.12). There was also no significant difference in the rate of falls in the previous three months between those who attended the 12-week and 24-week programmes (Incident rate ratio [IRR] 0.75 [95% CI 0.31 to 1.80], *p* = 0.52). Supplementary File S3 shows the additional analysis of falls rates across the study follow-up period (rather than follow-up data that only accounted for the most recent three months before final follow-up). This took account of data from 11 participants who attended 24-week programmes and had reported whether they had fallen in the past three months at both 12- and 24-week follow-up timepoints. No additional falls were recorded in these reports and, like the main analysis, no significant difference in falls rates between 12-week and 24-week programmes was found (IRR 1.10 [95% CI -0.76 to 1.58], *p* = 0.62).

**Table 3:** Data completeness summary

Age	Number of participants (%)	Number of provider organisations (out of 14) at which this information was collected from at least one participant
	1,434 (89.6%)	14
Ethnicity	630 (39.4%)	9
Index of multiple deprivation	1,311 (81.9%)	11
Baseline timed up and go	1,091 (68.1%)	14
12-week timed up and go*	348 (21.7%)	11
24-week timed up and go*	245 (15.3%)	7
Baseline falls in past 3 months	713 (44.5%)	9
12-week falls in past 3 months*	159 (9.9%)	6
24-week falls in past 3 months*	217 (13.6%)	5
Baseline Short FES-I	306 (19.1%)	10
12-week Short FES-I	145 (9.1%)	8
24-week Short FES-I	120 (7.5%)	6

*One of the provider organisations did not systematically do follow ups at standard times such as 12-weeks or 24-weeks and their follow data are not included in this table. The follow-up data collected at this organisation included: falls [*n* = 277] and timed up and go [*n* = 260]

The MI sensitivity analyses did not indicate a significant effect of programme duration upon TUG, which is likely to be reflective of these data not being missing at random. Otherwise, MI results were consistent with complete case analyses in finding 24- compared with 12-week programmes were associated with lower levels of concerns about falling, and no significant association between programme duration and the odds of having a fall (see Table 3). The sensitivity multivariable regression analyses that did not include ethnicity had findings consistent with the main analysis, except for the effect of programme duration upon TUG not being statistically significant in the sensitivity analysis (difference between means − 2.68 [95% CI -4.84 to 0.31], see Supplementary file S4).

Post-hoc power calculations indicated that adequate power (≥ 80%) to detect moderate differences in falls and in concerns about falling between the 12- and 24-week programmes was not achieved (see Supplementary file S2) (Table 4).

**Table 4:** Multilevel regression results for effect of whether the Falls Management Exercise (FaME) programme duration was 12 or 24 weeks on Short Falls Efficacy Scale-International (Short FES-I), falls and Timed Up and Go

Measure	Measurement time point	12-week duration *n* = 243	24-week duration *n* = 351	Primary (complete case) analysis				Multiple imputation (MI) analysis**	
				Unadjusted (except for baseline values for final follow-ups) differences between 12-week and 24-week programmes* (odds ratios for logistic, differences between means for linear, incident rate ratios for rate regressions (95% CI))	*p*	Adjusted† differences between 12-weeek and 24-week programmes* (odds ratios for logistic, differences between means for linear, incident rate ratios for rate regressions (95% CI))	*p*	Adjusted† differences between 12-weeek and 24-week programmes* (odds ratios for logistic, differences between means for linear, incident rate ratios for rate regressions (95% CI))	*p*
Logistic regression									
Number with a Short FES-I score ≥ 11 (*n* (proportion)	Baseline	61 of 78 (78.2%) [165]	37 of 90 (41.1%) [261]	0.21 (0.08 to 0.55)	0.001	0.32 (0.11 to 0.92)	0.034	0.19 (0.09 to 0.39)	< 0.001‡
	Final follow-up measurement	53 of 77 (68.8%) [166]	59 of 120 (49.2%) [231]	0.13 (0.02 to 0.74)	0.022	0.12 (0.02 to 0.69)	0.017	0.39 (0.16 to 0.97)	0.042‡
Number who had a fall in the past 3 months (*n* (proportion))	Baseline	43 of 76 (56.6%) [167]	104 of 279 (37.3%) [72]	2.19 (0.71 to 6.72)	0.17	2.04 (0.61 to 6.80)	0.24	1.96 (0.60 to 6.38)	0.27
	Final follow-up measurement	12 of 81 (14.8%) [162]	23 of 217 (10.6%) [134]	0.63 (0.20 to 1.91)	0.41	0.40 (0.12 to 1.26)	0.12	0.47 (0.16 to 1.33)	0.15
Linear regression									
Short FES-I scale (Mean ± SD)	Baseline	13.92 ± 4.16 [165]	10.83 ± 4.00 [261]	-2.99 (-4.45 to -1.53)	< 0.001	-2.68 (-4.60 to -0.75)	0.007	-3.11 (-4.41 to -1.82)	< 0.001
	Final follow-up measurement	13.22 ± 4.18 [166]	11.40 ± 4.31 [231]	-2.74 (-4.22 to -1.25)	< 0.001	-2.00 (-3.65 to -0.34)	0.018	-2.11 (-3.56 to -0.67)	0.004
Timed up and Go in seconds (Mean ± SD)	Baseline	15.40 ± 6.73 [17]	16.82 ± 8.29 [84]	-0.17 (-2.17 to 1.82)	0.87	-0.25 (-2.42 to 1.93)	0.82	-0.32 (-2.28 to 1.63)	0.75
	Final follow-up measurement	15.80 ± 8.44 [123]	15.14 ± 10.11 [106]	-1.41 (-3.57 to 0.75)	0.20	-3.31 (-5.97 to -0.65)	0.015	-1.91 (-4.10 to 0.28)	0.087
Rate regressions									
Falls (rate per person-year (*n* events))	Baseline	6.47 (123) [167]	3.93 (274) [72]	1.12 0.77 to 1.62)	0.57§	1.10 (0.76 to 1.58)	0.62§	0.52 (0.40 to 0.64)	< 0.001‡
	Final follow-up measurement	0.94 (19) [162]	0.65 (35) [134]	0.95 (0.39 to 2.35)	0.91§	0.75 0.31 to 1.80)	0.52	0.78 (0.36 to 1.68)	0.52‡

The models included organisation at level 2 and region at level 3

[ ] Missing values

*12-week duration group was the reference group

** for which the covariates gender, age and index of multiple deprivation were imputed

†The model adjusted for: age, gender, ethnicity, deprivation quintile and for follow-up measures, the relevant baseline score

‡A single level model was used (for complete case analyses when multilevel did not converge or for multiple imputation analysis due to non-availability of a STATA two-level multiple imputation count regression command)

§ A negative binomial model was used due to evidence of overdispersion

## Discussion

This study presents an evaluation of FaME, implemented across three UK regions. Our multivariable analysis investigated differences between participants in 12-week and 24-week FaME programmes. Those who had attended 24- rather than 12-week FaME programmes had significantly quicker TUG times, by 3.31 s, in regression analyses (though this result was not replicated in MI analysis or sensitivity analysis that did not include ethnicity in the model). Those in the longer programmes also had 88% lower odds of having ‘high level concerns’ about falling and had a mean Short FES-I score 2.0 points lower (indicating lower concerns). There was no significant difference between the 12- and 24-week programmes in the proportion who reported to have fallen, or the number of falls that had occurred, in the 3 months prior to follow-up, however these analyses were not adequately powered to detect a difference. The TUG findings reported in the present study are similar to those in a published evaluation of FaME when it was implemented across a single UK region [9]. In the previous single-region study, a reduction of 13% was reported and the median TUG at the end of the study was 11.36 [9]. In the present study, the proportional reduction in median TUG was 15.8% (from 9.5 to 8.0). There were, however, some differences between the studies in the reported levels of concerns about falling. The likelihood of having ‘higher’ concerns about falling at the end of the present study (50.7%) was higher than that reported in the single-region study (29.6%) [9]. We cannot be certain about the reason for this difference, but participants of the present study were around 5 years older on average compared to the single-region study, which may explain some of the variation. It has also been reported that since the COVID-19 pandemic, older adults’ perceptions of falls risk [18] and levels of frailty may have increased [19]. These population level changes may have also affected our findings.

When considering our multivariable findings comparing Short FES-I scores between 12- and 24-week programmes, it should be noted that at baseline the 24-week duration group also had significantly lower odds (by 68%) of having ‘higher concerns’ about falling and had significantly lower (by 2.68 points) Short FES-I scores. We did not detect other differences between these two groups at baseline that offer clear explanations for these differences (see Supplementary Table 1). Concerns about falling represent an important barrier to physical activity [5, 20] and are important independent predictors of future falls [20]. We know that to retain benefits of FaME over the longer-term people have to remain physically active and continue with strength and balance exercise [21, 22], therefore these additional benefits at 24 week programmes are likely to confer longer term benefits than 12 week programmes. Furthermore, it can take 6 months to adopt a behaviour [23]. The longer programmes may therefore be more likely to lead to lasting behavioural changes in the form of increased activity levels that reduce the risk of falling [24]. However, we did not have sufficient physical activity information gathered by our providers to be able to test whether participants of 24-week programmes were more physically active. It would be useful for this to be measured by FaME programmes in future.

Our univariate before-and-after tests indicated that, irrespective of programme duration, participants had a significantly lower odds (a reduction of 84%) of reporting at least one fall in the previous three months at the end of the programme than at the start. They also had statistically significantly improved TUG times, with the difference for paired medians being 1.0 s between the start and end of the programme. No significant change was seen in short FES-I scores between baseline and final follow-up. It is important to consider, however, that follow-up data were only available for 45–54% of participants with baseline data and it may be that this introduced an attrition bias if these data were not missing at random.

Our univariate finding of reduced odds of a fall at the end compared to the start of the programme is consistent with findings from previous ‘real-life’ reductions in falls seen in 24-week programmes [9]. Evidence from previous research suggests that the univariate TUG finding – reflecting a reduction from a group median of 9.5 to 8.0 s - represents a clinically significant improvement [25]. Although no minimal detectable change (MDC) data are available for TUG in older adults, in people with Parkinson’s Disease it is 3.5 s [26] and in people with osteoarthritis it is 1.1 s [25].

In the present study and the previous single-region FaME Evaluation [9], the numbers of men and those with an ethnicity other than White were similarly low. This limits the generalisability of the findings to these groups. Most published studies on falls prevention exercise are carried out in women [27] and the relationship between ethnicity and risk of falling is not well understood, especially for the UK [28]. More work is therefore needed to attract men and people of ethnicities other than White British into falls prevention exercise programmes. We note that amongst participants with data at 12-week follow-up, 38.7% had postcodes in the three most deprived IMD deciles compared to 29.6% of those who attended 24-week programmes (see Table 1). We do not know whether this difference was related to any differences in funding between programmes in areas of different levels of deprivation or possibly of people in more deprived areas having less opportunity to attend the longer programmes.

Study strengths include the real-world setting, which comprised multiple sites across three UK regions and data collected by the services. Our multilevel analysis accounted for the different regions and sites and controlled for age, gender, deprivation and ethnicity. Study limitations include there not having been adequate data collected by providers on participant comorbidities or medications for these factors to be included in our multivariate analyses. There were also large amounts of missing covariate and follow-up data, and this varied between provider organisations (though we conducted MI analysis to address missing covariate data and were able to detect significant differences in TUG and concerns about falling between 12- and 24-week programmes in the compete case analyses despite the missing data).

Post-hoc power calculations indicated that the study lacked adequate power to detect clinically important differences in falls and in concerns about falling between the 12- and 24-week programmes. This may provide an explanation as to why we detected a significant difference in functional outcomes between programmes lasting 12 and 24 weeks but did not detect a difference in falls. It is also possible that individuals who found the FaME classes particularly challenging – potentially due to experiencing frailty, poorer health or an injury due to falling – were more likely to cease their attendance early, without their final follow-up information being recorded. An impact of this would have been to inflate the positive before-and-after comparison findings (compared to, for example, a trial with an intention to treat analysis). It is not possible to infer from our data, however, the extent to which early participant withdrawal from the programmes explains the quantity of missing follow-up information. Moreover, a recently published study of the fidelity of the delivery of FaME indicates variability between delivery sites in how proactive site staff were in ensuring follow-up assessment data were captured [29].

It is suggested here that future FaME evaluations of service outcomes should promote more consistent and systematic data collection. Additionally, services should have specifically focussed strategies to include men and those from ethnic minority groups. It should also be noted that the deprivation covariate we used was area-level based on postcode rather than individual-level. Our use of retrospectively reported falls data may have been less accurate than collecting data prospectively with falls calendars [30], but this was weighed against the benefits of there being reduced attrition associated with this approach, compared with prospective diary methods [31], especially given research indicates both methods can give similar results [32].

## Conclusions

FaME, delivered over three regions, was associated with benefits at 12 and 24 weeks. These included improved functional mobility and reduced falls. Those whose programme lasted 24 rather than 12 weeks experienced greater improvements in functional mobility and were less likely to be concerned about falling. We recommend that in order to achieve the best outcomes, FaME should be commissioned as the 24-week programme. However, if 12-week programmes are commissioned there must be an additional effort to transition people on to other standing strength and balance exercise opportunities to ensure ongoing beneficial outcomes and to establish longer-term behaviour change.

## Supplementary Information

Supplementary Material 1.

## Data Availability

Data analysed in this report are not available for sharing. The data were shared with the research team as part of information sharing agreements and permission to make the data available beyond the research team was not granted. Study documentation is available upon reasonable request from the corresponding author (Michael J Taylor, [michael.taylor6@nottingham.ac.uk](mailto: michael.taylor6@nottingham.ac.uk) ).

## References

[1] Cuevas-Trisan R. Balance problems and fall risks in the elderly. Phys Med Rehabil Clin. 2017;28(4):727–37. 10.1016/j.pmr.2017.06.006 29031339

[2] Haagsma JA, Olij BF, Majdan M, Van Beeck EF, Vos T, Castle CD, et al. Falls in older aged adults in 22 European countries: incidence, mortality and burden of disease from 1990 to 2017. Inj Prev. 2020;26(Suppl 2):i67–74. 32111726 10.1136/injuryprev-2019-043347 PMC7571349

[3] Su FY, Fu ML, Zhao QH, Huang HH, Luo D, Xiao MZ. Analysis of hospitalization costs related to fall injuries in elderly patients. World J Clin cases. 2021;9(6):1271. 33644194 10.12998/wjcc.v9.i6.1271 PMC7896694

[4] Department of Health. and Social Care. Fingertips. Public health profiles. [Internet]. 2024 [cited 2024 Dec 23]. Available from: https://fingertips.phe.org.uk/search/falls.

[5] Montero-Odasso M, Van Der Velde N, Martin FC, Petrovic M, Tan MP, Ryg J, et al. World guidelines for falls prevention and management for older adults: a global initiative. Age Ageing. 2022;51(9):afac205. 36178003 10.1093/ageing/afac205 PMC9523684

[6] Iliffe S, Kendrick D, Morris R, Masud T, Gage H, Skelton D, et al. Multicentre cluster randomised trial comparing a community group exercise programme and home-based exercise with usual care for people aged 65 years and over in primary care. Health Technol Assess. 2014;18(49):vii. 25098959 10.3310/hta18490 PMC4781144

[7] Skelton D, Dinan S, Campbell M, Rutherford O. Tailored group exercise (Falls Management Exercise—FaME) reduces falls in community-dwelling older frequent fallers (an RCT). Age Ageing. 2005;34(6):636–9. 16267192 10.1093/ageing/afi174

[8] Iliffe S, Kendrick D, Morris R, Griffin M, Haworth D, Carpenter H, et al. Promoting physical activity in older people in general practice: ProAct65 + cluster randomised controlled trial. Br J Gen Pract. 2015;65(640):e731–8. 26500320 10.3399/bjgp15X687361 PMC4617267

[9] Orton E, Audsley S, Coupland C, Gladman JRF, Iliffe S, Lafond N, et al. Real world effectiveness of the Falls Management Exercise (FaME) programme: an implementation study. Age Ageing. 2021;50(4):1290–7. 33529311 10.1093/ageing/afaa288

[10] Skelton DA, Dinan SM. Exercise for falls management: Rationale for an exercise programme aimed at reducing postural instability. Physiother Theory Pract. 1999;15(2):105–20.

[11] Podsiadlo D, Richardson S. The timed Up & Go: a test of basic functional mobility for frail elderly persons. J Am Geriatr Soc. 1991;39(2):142–8. 1991946 10.1111/j.1532-5415.1991.tb01616.x

[12] Kempen GIJM, Yardley L, Van Haastregt JCM, Zijlstra GAR, Beyer N, Hauer K, et al. The Short FES-I: a shortened version of the falls efficacy scale-international to assess fear of falling. Age Ageing. 2008;37(1):45–50. 18032400 10.1093/ageing/afm157

[13] Delbaere K, Close JCT, Mikolaizak AS, Sachdev PS, Brodaty H, Lord SR. The falls efficacy scale international (FES-I). A comprehensive longitudinal validation study. Age Ageing. 2010;39(2):210–6. 20061508 10.1093/ageing/afp225

[14] Geng Y, Lo JC, Brickner L, Gordon NP. Racial-ethnic differences in fall prevalence among older women: a cross-sectional survey study. BMC Geriatr. 2017;17:1–7. 28284206 10.1186/s12877-017-0447-y PMC5346231

[15] Johnson NA, Dias JJ. The effect of social deprivation on fragility fracture of the distal radius. Injury. 2019;50(6):1232–6. 31076143 10.1016/j.injury.2019.04.025

[16] GOV.UK. List of ethnic groups. [Internet]. [cited 2024 May 14]. Available from: https://www.ethnicity-facts-figures.service.gov.uk/style-guide/ethnic-groups/.

[17] National Statistics. English indices of deprivation 2019. [Internet]. 2019 [cited 2022 Apr 12]. Available from: https://www.gov.uk/government/statistics/english-indices-of-deprivation-2019.

[18] Kiyoshi-Teo H, Izumi S, Stoyles S, McMahon SK. Older adults’ biobehavioral fall risks were affected by the COVID-19 pandemic: Lessons learned for future fall prevention research to incorporate multilevel perspectives. Innov aging. 2022;6(6):igac033. 36161144 10.1093/geroni/igac033 PMC9495495

[19] König M, Gollasch M, Komleva Y. Frailty after COVID-19: The wave after? Aging Med. 2023;6(3):307–16. 10.1002/agm2.12258 PMC10498835 37711259

[20] Ellmers TJ, Ventre JP, Freiberger E, Hauer K, Hogan DB, Lim ML et al. Does concern about falling predict future falls in older adults? A systematic review and meta-analysis. Age Ageing. 2025;54(4):afaf089. 10.1093/ageing/afaf089 PMC11976718 40197783

[21] Maula A, LaFond N, Orton E, Iliffe S, Audsley S, Vedhara K, et al. Use it or lose it: a qualitative study of the maintenance of physical activity in older adults. BMC Geriatr. 2019;19(1):349. 31830900 10.1186/s12877-019-1366-x PMC6909612

[22] Manley AF. Physical activity and health: A report of the Surgeon General.US Department of Health and Human Services, Public HealthService, CDC, National Center for Chronic Disease Prevention and Health Promotion; 1996.

[23] Stiggelbout M, Hopman-Rock M, Crone M, Lechner L, Van Mechelen W. Predicting older adults’ maintenance in exercise participation using an integrated social psychological model. Health Educ Res. 2006;21(1):1–14. 15980075 10.1093/her/cyh037

[24] Hawley-Hague H, Horne M, Campbell M, Demack S, Skelton DA, Todd C. Multiple levels of influence on older adults’ attendance and adherence to community exercise classes. Gerontologist. 2014;54(4):599–610. 23899623 10.1093/geront/gnt075 PMC4102320

[25] Alghadir A, Anwer S, Brismée JM. The reliability and minimal detectable change of Timed Up and Go test in individuals with grade 1–3 knee osteoarthritis. BMC Musculoskelet Disord. 2015;16:1–7. 26223312 10.1186/s12891-015-0637-8 PMC4520098

[26] Huang SL, Hsieh CL, Wu RM, Tai CH, Lin CH, Lu WS. Minimal detectable change of the timed up & go test and the dynamic gait index in people with Parkinson disease. Phys Ther. 2011;91(1):114–21. 20947672 10.2522/ptj.20090126

[27] Sandlund M, Skelton DA, Pohl P, Ahlgren C, Melander-Wikman A, Lundin-Olsson L. Gender perspectives on views and preferences of older people on exercise to prevent falls: a systematic mixed studies. 2017. 10.1186/s12877-017-0451-2 PMC5316178 28212622

[28] Wehner-Hewson N, Watts P, Buscombe R, Bourne N, Hewson D. Racial and ethnic differences in falls among older adults: a systematic review and meta-analysis. J racial Ethn Heal disparities. 2022;9(6):2427–40. 10.1007/s40615-021-01179-1 PMC9633486 34786654

[29] Manning F, Ventre JP, Brough G, Hawley-Hague H, Hulme C, Kendrick D, et al. Mediators implementation and delivery: the falls management exercise programme (FaME). BMC Health Serv Res. 2025;25(1):1–15. 41126178 10.1186/s12913-025-13550-7 PMC12542040

[30] Hannan MT, Gagnon MM, Aneja J, Jones RN, Cupples LA, Lipsitz LA, et al. Optimizing the tracking of falls in studies of older participants: comparison of quarterly telephone recall with monthly falls calendars in the MOBILIZE Boston Study. Am J Epidemiol. 2010;171(9):1031–6. 20360242 10.1093/aje/kwq024 PMC2877474

[31] Griffin J, Lall R, Bruce J, Withers E, Finnegan S, Lamb SE, et al. Comparison of alternative falls data collection methods in the Prevention of Falls Injury Trial (PreFIT). J Clin Epidemiol. 2019;106:32–40. 30266633 10.1016/j.jclinepi.2018.09.006

[32] Rapp K, Freiberger E, Todd C, Klenk J, Becker C, Denkinger M, et al. Fall incidence in Germany: results of two population-based studies, and comparison of retrospective and prospective falls data collection methods. BMC Geriatr. 2014;14(1):105. 25241278 10.1186/1471-2318-14-105 PMC4179843

