# Supplementary material for: Evaluation of the Falls Management Exercise (FaME) programme implemented across three United Kingdom regions: a prospective cohort study
Source: BMC Public Health. 2026 Mar 6;26:1211. doi: 10.1186/s12889-026-26893-5 (PMC13081467; doi:10.1186/s12889-026-26893-5)
Supplement: Supplementary file 1 — Supplementary Material 1. [file 12889_2026_26893_MOESM1_ESM.docx]

Supplementary file S1: characteristics of participants included in complete case regression analyses of data at final follow-up

|  | **Short FES-I scale/binary (whether score of at least 11)** | | **Falls count/binary (whether a fall in previous 3 months)** | | **Timed Up-and-Go** | |
| --- | --- | --- | --- | --- | --- | --- |
|  | ***12-week duration***  ***n* = 33** | ***24-week duration***  ***n* = 65** | ***12-week duration***  ***n* = 61** | ***24-week duration***  ***n* = 176** | ***12-week duration***  ***n* = 87** | ***24-week duration***  ***n* = 190** |
| **Median (Interquartile range)** |  |  |  |  |  |  |
| Age, median (IQR) | 77.5  (73.5 to 83.5) | 81.5  (77.5 to 86.5) | 80.5  (73.5 to 85.5) | 78.5  (74.5 to 83.5) | 73.5  (67.5 to 81.5) | 79.5  (75.5 to 84.5) |
| **N (% by group)** |  |  |  |  |  |  |
| Region:  Devon  East Midlands  Greater Manchester | 27 (81.8%)  0 (0%)  6 (18.2%) | 12 (18.5%)  0 (0%)  53 (81.5%) | 22 (36.0%)  0 (0.0%)  39 (63.9%) | 6 (3.4%)  133 (75.6%)  37 (21.0%) | 23 (26.4%)  0 (0%)  64 (73.6%) | 12 (6.3%)  94 (49.5%)  84 (44.2%) |
| Gender:  Female  Male | 26 (78.8%)  7 (21.2%) | 48 (73.9%)  17 (26.2%) | 46 (75.4%)  15 (24.6%) | 123 (69.9%)  53 (30.1%) | 57 (65.5%)  30 (34.5%) | 136 (71.6%)  54 (28.4%) |
| Ethnicity  Asian  Black  Mixed  White  Other ethnicity  Unknown or missing | 1 (3.0%)  0 (0%)  0 (0%)  11 (33.3%)  0 (0%)  21 (63.6%) | 1 (1.5%)  0 (0%)  0 (0%)  47 (72.3%)  0 (0%)  17 (26.2%) | 0 (0%)  1 (1.6%)  0 (0%)  38 (62.3%)  0 (0%)  22 (36.1%) | 5 (2.8%)  0 (0%)  0 (0%)  154 (87.5%)  1 (0.6%)  16 (9.1%) | 3 (3.5%)  0 (0%)  0 (0%)  25 (28.7%)  0 (0%)  59 (67.8%) | 5 (2.6%)  0 (0%)  0 (0%)  169 (89.0%)  1 (0.5%)  16 (8.4%) |
| IMD  1 (most deprived)  2  3  4  5 (least deprived) | 1 (3.0%)  4 (12.1%)  14 (42.4%)  9 (27.3%)  5 (15.2%) | 12 (18.5%)  11 (16.9%)  15 (23.1%)  18 (27.7%)  9 (13.9%) | 8 (13.1 %)  14 (23.0%)  19 (31.2%)  15 (24.6%)  5 (8.2%) | 8 (4.6%)  18 (10.2%)  33 (18.8%)  49 (27.8%)  68 (38.6%) | 35 (40.2%)  12 (13.8%)  16 (18.4%)  16 (18.4%)  8 (9.2%) | 18 (9.5%)  25 (13.2%)  37 (19.5%)  65 (34.2%)  45 (23.7%) |

Supplementary file S2: Post hoc power calculations

**Post-hoc power estimates**

**Univariable tests comparing baseline and final follow-up values**

The power (1-*β*) achieved for the univariate Short FES-I paired significance McNemar’s test for the binary outcome was 0.36 (with an odds ratio of 0.583, alpha of 0.05, and sample size of 142, with 38 discordant pairs). To achieve a power of 0.80 with the same odds ratio and proportion of discordant pairs, a total sample size of 439 would have been required.

For the Short FES-I scale outcomes, the power achieved was 0.47 (based on a paired samples *t* test with mean difference between baseline and final follow-up scores of -0.606 and standard deviation of 3.81). 313 participants would have been needed to be included to achieve a power of 0.80 with the same effect size.

**Tests comparing programme durations**

Comparing the effect of programme duration (12 or 24 weeks) upon the proportions of people who experienced a fall in the previous 3 months, the achieved power was 0.19 (a 2 tailed Z test comparing 23/217 (10.6%) for 24 weeks and 12/81 (14.8%) for 12 weeks). To achieve a power of 0.8 with the same proportions reporting falls and the same ratio of group sizes a total of 2423 participants would have been required.

Supplementary table S3: Falls sensitivity analysis

| **Measure** | **Measurement time point** | **12-week duration** | **24-week duration** | **Primary (complete case) analysis** | | | | **Multiple imputation (MI) analysis**** | |
| --- | --- | --- | --- | --- | --- | --- | --- | --- | --- |
|  |  |  |  | **Unadjusted (except for baseline values for final follow-ups) incident rate ratio between 12-weeek and 24-week programmes (95% CI)*** | ***p*** | **Adjusted† incident rate ratios odds ratio between 12-weeek and 24-week programmes (95% CI)*** | ***p*** | **Adjusted incident rate ratios between 12-weeek and 24-week programmes (95% CI)*** | ***p*** |
| Falls (rate per person- year (n events)) | Final follow-up | 0.94 (19) | 0.48 (35) | 0.56  (0.28 to 10.66) | 0.35 | 1.10  (-0.76 to 1.58) | 0.62‡ | 0.69  (0.32 to 1.48) | 0.34 |

*12-week duration group was the reference group

** for which the covariates gender, age and index of multiple deprivation were imputed

†The model controlled for: age, gender, ethnicity, deprivation quintile and for follow-up measures, the relevant baseline score.

‡A single level model was used (for complete case analyses when multilevel did not converge or for multiple imputation analysis due to non-availability of a STATA two-level multiple imputation count regression command)

Supplementary table S4: Sensitivity analysis findings for multilevel regression that did not include ethnicity in the model showing the effect of whether the Falls Management Exercise (FaME) programme duration was 12 or 24 weeks on Short Falls Efficacy Scale-International (Short FES-I), falls and Timed Up and Go

| **Measure** | **Measurement time point** | **12-week duration**  ***n* = 243** | **24-week duration**  ***n* = 351** | **Primary (complete case) analysis** | | | | **Multiple imputation (MI) analysis**** | |
| --- | --- | --- | --- | --- | --- | --- | --- | --- | --- |
|  |  |  |  | **Unadjusted (except for baseline values for final follow-ups) differences between 12-week and 24-week programmes* (odds ratios for logistic, differences between means for linear, incident rate ratios for rate regressions (95% CI))** | ***p*** | **Adjusted† differences between 12-weeek and 24-week programmes* (odds ratios for logistic, differences between means for linear, incident rate ratios for rate regressions (95% CI))** | ***p*** | **Adjusted† differences between 12-weeek and 24-week programmes* (odds ratios for logistic, differences between means for linear, incident rate ratios for rate regressions (95% CI))** | ***p*** |
| **Logistic regression** | | | | | | | | | |
| Number with a Short FES-I score ≥11  (*n* (proportion) | Baseline | 61 of 78 (78.2%) [165] | 37 of 90 (41.1%) [261] | 0.21 (0.08 to 0.55) | 0.001 | 0.28  (0.10 to 0.81) | .019 | 0.19  (0.09 to 0.39) | <.001‡ |
|  | Final follow-up measurement | 53 of 77 (68.8%) [166] | 59 of 120 (49.2%) [231] | 0.13  (0.02 to 0.74) | 0.022 | 0.12  (0.02 to 0.71) | 0.020 | 0.40  (0.17 to 0.96) | 0.041‡ |
| Number who had a fall in the past 3 months  (*n* (proportion)) | Baseline | 43 of 76 (56.6%) [167] | 104 of 279 (37.3%) [72] | 2.19  (0.71 to 6.72) | 0.17 | 2.15  (0.67 to 6.93) | 0.20 | 2.17  (0.69 to 6.77) | 0.18 |
|  | Final follow-up measurement | 12 of 81 (14.8%) [162] | 23 of 217 (10.6%) [134] | 0.63  (0.20 to 1.91) | 0.41 | 0.39  (0.14 to 1.09) | 0.07 | 0.47  (0.19 to 1.16) | 0.10 |
| **Linear regression** | | | | | | | | | |
| Short FES-I scale (Mean±SD) | Baseline | 13.92±4.16 [165] | 10.83±4.00 [261] | -2.99  (-4.45 to -1.53) | <.001 | -2.53  (-4.34 to -0.72) | 0.006 | -2.93  (-4.22 to -1.63) | <.001 |
|  | Final follow-up measurement | 13.22±4.18 [166] | 11.40±4.31 [231] | -2.74  (-4.22 to -1.25) | <.001 | -2.80  (-4.15 to -0.40) | 0.017 | -1.96  (-3.38 to -0.53) | .007 |
| Timed up and Go in seconds (Mean±SD) | Baseline | 15.40±6.73 [17] | 16.82±8.29 [84] | -0.17  (-2.17 to 1.82) | 0.87 | -0.17  (-2.35 to 2.01) | 0.88 | -0.31  (-2.26 to 1.64) | 0.75 |
|  | Final follow-up measurement | 15.80±8.44 [123] | 15.14±10.11 [106] | -1.41  (-3.57 to 0.75) | 0.20 | -2.68  (-4.84 to 0.31) | 0.08 | -1.57  (-3.65 to 0.51) | 0.14 |
| **Rate regressions** | | | | | | | | | |
| Falls (rate per person-year (n events)) | Baseline | 6.47 (123) [167] | 3.93 (274) [72] | 1.12  (0.77 to 1.62) | 0.57§ | 1.11  (0.78 to 1.59) | §0.56 | 0.67  (0.54 to 0.84) | <.001 |
|  | Final follow-up measurement | 0.94 (19) [162] | 0.65 (35) [134] | 0.95  (0.39 to 2.35) | 0.91§ | 0.67  (0.31 to 1.49) | 0.33 | 0.67  (0.34 to 1.31) | .24 |

The models included organisation at level 2 and region at level 3

[ ] Missing values

*12-week duration group was the reference group

** for which the covariates gender, age and index of multiple deprivation were imputed

†The model adjusted for: age, gender, deprivation quintile and for follow-up measures, the relevant baseline score.

‡A single level model was used (for complete case analyses when multilevel did not converge or for multiple imputation analysis due to non-availability of a STATA two-level multiple imputation count regression command)

§ A negative binomial model was used due to evidence of overdispersion
